# BANNMDA: a computational model for predicting potential microbe–drug associations based on bilinear attention networks and nuclear norm minimization

**DOI:** 10.3389/fmicb.2024.1497886

**Published:** 2025-01-22

**Authors:** Mingmin Liang, Xianzhi Liu, Juncai Li, Qijia Chen, Bin Zeng, Zhong Wang, Jing Li, Lei Wang

**Affiliations:** ^1^School of Intelligent Equipment, Hunan Vocational College of Electronic and Technology, Changsha, China; ^2^School of Information Engineering, Hunan Vocational College of Electronic and Technology, Changsha, China; ^3^School of Humanities and Education, Hunan Vocational College of Electronic and Technology, Changsha, China; ^4^Big Data Innovation and Entrepreneurship Education Center of Hunan Province, Changsha University, Changsha, China

**Keywords:** computational model, microbe–drug associations, bilinear attention networks, nuclear norm minimization, prediction

## Abstract

**Introduction:**

Predicting potential associations between microbes and drugs is crucial for advancing pharmaceutical research and development. In this manuscript, we introduced an innovative computational model named BANNMDA by integrating Bilinear Attention Networks(BAN) with the Nuclear Norm Minimization (NNM) to uncover hidden connections between microbes and drugs.

**Methods:**

In BANNMDA, we initially constructed a heterogeneous microbe-drug network by combining multiple drug and microbe similarity metrics with known microbe-drug relationships. Subsequently, we applied both BAN and NNM to compute predicted scores of potential microbe-drug associations. Finally, we implemented 5-fold cross-validation frameworks to evaluate the prediction performance of BANNMDA.

**Results and discussion:**

The experimental results indicated that BANNMDA outperformed state-of-the-art competitive methods. We conducted case studies on well-known drugs such as the Amoxicillin and Ceftazidime, as well as on pathogens such as *Bacillus cereus* and Influenza A virus, to further evaluate the efficacy of BANNMDA, and experimental outcomes showed that there were 9 out of the top 10 predicted drugs, along with 8 and 9 out of the top 10 predicted microbes having been corroborated by relevant literatures. These findings underscored the capability of BANNMDA to achieve commendable predictive accuracy.

## Introduction

Microorganisms are tiny, structurally simple, and widely distributed organisms, including bacteria, viruses, and fungi. They are closely related to human health, offering both benefits and potential risks (Human Microbiome Project Consortium, 2012; Cheng et al., 2020). Various organs of the human body are inhabited by them and are even covered by them (Gill et al., 2006). These microorganisms play a role not only in promoting the absorption of food and maintaining intestinal health but also in effectively regulating the host’s mucosal and systemic immune systems by adjusting the balance of the gut microbiota (Ventura et al., 2009; Sommer and Bäckhed, 2013). In the intestinal environment, these microorganisms are interdependent and mutually beneficial. When the balance of the gut microbiota is disrupted, it can lead to a variety of diseases, including obesity (Ley et al., 2006), inflammatory bowel disease (Durack and Lynch, 2019), and cancer (Schwabe and Jobin, 2013). In addition, a multitude of studies have confirmed that there is a significant interaction between microorganisms and drugs during the drug treatment process (Human Microbiome Project Consortium, 2012; McCoubrey et al., 2022; Zhang et al., 2023). Therefore, a deep understanding of the relationship between microorganisms and drugs is crucial for the effective treatment of diseases.

Through in-depth biological research, humanity has uncovered key connections between drugs and microbes. However, biological experiments often require a significant investment of human resources, materials, and time, which may limit further in-depth research. To overcome the limitations of biological studies, the application of computational methods has been increasing in recent years, driven by the rapid development of related research tools. These computational methods are dedicated to predicting the interactions between drugs and microbes (Wang et al., 2022). Concurrently, databases of microbe–drug associations that have been experimentally validated, such as MDAD (Sun et al., 2018) (Doi: figshare.com/articles/dataset/MDAD__/24798456) and aBiofilm (Rajput et al., 2018) (Doi: figshare.com/articles/dataset/aBiofilm_dataset/28045016), have also been established, providing valuable data resources for research. For instance, Zhu et al. (2022) have introduced NNAN, a method that utilizes a nearest-neighbor information aggregator and a feature attention module to identify correlations between microbes and drugs. Deng et al. (2022) have proposed a new method, Graph2MDA, which utilizes a Variational Graph Auto-Encoder (VGAE) to predict associations between microbes and drugs. In an effort to infer novel relationships between microbes and drugs, Yang et al. (2022) have proposed a multi-kernel fusion model based on Graph Convolutional Networks (GCN), known as MKGNN. Tian et al. (2023) have crafted a contrastive learning model for predicting connections between microbes and drugs, called SCSMDA. Tan et al. (2022) have developed a computational technique based on graph attention networks and sparse autoencoders for predicting potential microbe–drug correlations, named GSAMDA. Ma et al. (2023) have developed a predictive model for microbe–drug interactions that integrate the capabilities of Graph Attention Networks (GAT) with the image-processing prowess of Convolutional Neural Networks (CNN).

**Figure 1:**
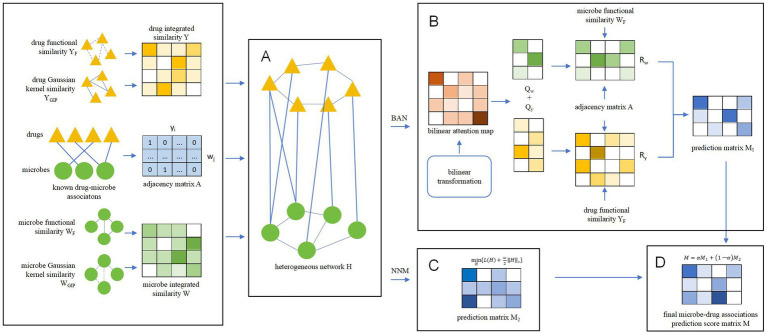
Overall structure diagram of BANNMDA. **(A)** The heterogeneous microbe–drug network was established by amalgamating the microbe similarity network, the drug similarity network, and known associations between microbes and drugs. **(B)** Predicting potential microbe–drug associations by BAN. **(C)** Predicting potential microbe–drug associations by NNM. **(D)** Predicting the final scores of potential microbe–drug associations.

Inspired by Liu et al. (2023) and Bai et al. (2023), we designed a novel prediction model called BANNMDA based on the bilinear attention network and kernel norm minimization to accurately infer potential associations between microorganisms and drugs. As illustrated in Figure 1, the principal contributions of BANNMDA include:

- A novel heterogeneous microbe–drug network *H* was established by amalgamating the microbe similarity network, the drug similarity network, and known associations between microbes and drugs.
- To forecast potential microbe–drug association scores more accurately, we would first use a BAN-based autoencoder alongside the nuclear norm minimization technique on *N* to calculate two predicted scores for potential microbe–drug associations, respectively. Then, we would further combine these two predicted scores through a weighted average to derive the conclusive outcomes.

## Materials and methods

### Data sources

To assess the predictive performance of the BANNMDA model, we selected the MDAD dataset. The MDAD dataset, compiled by Sun et al. (2018), is an extensive compilation of microbe–drug associations. It was sourced from various drug databases such as TTD and DrugBank, along with extensive literature, resulting in a database of 1,373 drugs and 173 microbes connected by 2,470 associations after removing redundant entries. Table 1 provides specific statistical data for the MDAD dataset.

**Table 1:** Specific statistical data for MDAD datasets.

Dataset	Microbes	Drugs	Associations
MDAD	173	1,373	2,470

### Methods

#### Microbe–drug adjacency matrix

An adjacency matrix, designated as $A\in R^{n_{y}\times n_{w}}$ , is initially constructed. This matrix captures the interactions between a set of drugs (denoted by $n_{y}$ ) and microbes (denoted by $n_{w}$ ). The matrix is populated such that each entry is marked 1 if a relationship is established between a specific drug $y_{i}$ and a particular microbe $w_{j}$ , and 0 otherwise. As the Equation 1 shown.


(1) $$A_{ij}=\{\begin{array}{c} 1,\mathrm{if}\;y_{i}\;\mathrm{associats with}\;w_{j}\\ 0,\mathrm{otherwise} \end{array}$$

#### Microbe/drug Gaussian kernel similarity

The Gaussian kernel similarity is calculated by using the Gaussian kernel function, which is a widely used kernel function for measuring the similarity between elements. In the field of microbe–drug association prediction, the Gaussian kernel similarity is one of the most popular methods for measuring similarity between microbes and drugs, which is based on the assumption that two similar microbes will exhibit similar interactive and non-interactive relationships with the same drug.

The Gaussian kernel similarity $Y_{GIP}\left(y_{i},y_{j}\right)\in R^{n_{y}\times n_{y}}$ between drugs $y_{i}$ and $y_{j}$ , can be calculated by using the Equation 2:


(2) $$Y_{GIP}=\exp\left(-\gamma_{y}{\left\|A\left(y_{i}\right)-A\left(y_{j}\right)\right\|}^{2}\right)$$

Certainly, let us clarify the role of $\left\|A\left(y_{i}\right)-A\left(y_{j}\right)\right\|$ in the context of Gaussian kernel similarity, particularly as it pertains to the Euclidean distance between two drugs. The parameter $\gamma_{y}$ plays a crucial role in determining the influence of the distance between feature points. Equation 3 shows how it works:


(3) $$\gamma_{y}=1/\left(\frac{1}{n_{y}}\overset{n_{y}}{\underset{i=1}{\sum }}{\left\|A\left(y_{i}\right)\right\|}^{2}\right)$$

The Gaussian kernel similarity $W_{GIP}\left(w_{i},w_{j}\right)\in R^{n_{w}\times n_{w}}$ can be similarly applied to measure the similarity between microbes. Equations 4, Equation 5 show how to get the Gaussian kernel similarity:


(4) $$W_{GIP}=\exp\left(-\gamma_{w}{\left\|A\left(w_{i}\right)-A\left(w_{j}\right)\right\|}^{2}\right)$$

(5) $$\gamma_{w}=1/\left(\frac{1}{n_{w}}\overset{n_{w}}{\underset{i=1}{\sum }}\|A\left(w_{i}\right)\|{∥}^{2}\right)$$

#### Microbe/drug functional similarity

The microbe functional similarity is determined by leveraging the Kamneva tool (Kamneva, 2017), which is grounded in the analysis of microbial gene families. The process begins with the construction of a microbial protein–protein functional association network using the comprehensive STRING (Szklarczyk et al., 2019) dataset, which provides a rich collection of gene functional networks related to microbes. In this network, nodes represent gene families encoded by the genome, and edges signify genetic neighborhood scores. To evaluate the functional similarities between microbes, a matrix $W_{F}\in R^{n_{w}\times n_{w}}$ is crafted using the Kamneva tool, which calculates the similarity by comparing the score of the edges between two microbes to the sum of all link scores corresponding to their microbial gene families.

Furthermore, the SIMCOMP (Hattori et al., 2010) tool harnesses the chemical structures and molecular formulas of drugs to quantify their structural similarity. The core of this method is to realize the automated matching of nodes and edges across two chemical structure diagrams by software algorithms. By identifying the most extensive common substructure, this method can assess and calculate the similarities between different drug frameworks. Based on this method, a drug functional similarity matrix $Y_{F}\in R^{n_{y}\times n_{y}}$ can be constructed.

#### Microbe/drug integrated similarities

It is essential to acknowledge that not all microbes can be effectively compared in terms of functional similarity. To address this, we have utilized both the structural similarity and the Gaussian kernel similarity of microbes. By combining these metrics, we have successfully created a novel matrix $W\in R^{n_{w}\times n_{w}}$ by using Equation 6. This integrated matrix provides a more comprehensive and nuanced assessment of microbe similarities, offering valuable insights into their complex relationships.


(6) $$W=\{\begin{array}{c} \left(W_{GIP}+W_{F}\right)/2,\mathrm{if}\;W_{F}\neq 0\\ W_{GIP},\mathrm{if}\;W_{F}=0 \end{array}$$

Similarly, the drug matrix can be obtained as Equation 7:


(7) $$Y=\{\begin{array}{c} \left(Y_{GIP}+Y_{F}\right)/2,\mathrm{if}\;Y_{F}\neq 0\\ Y_{GIP},\mathrm{if}\;Y_{F}=0 \end{array}$$

### Constructing the heterogeneous network H

By integrating the microbe–drug adjacency matrix with the drug functional similarity matrix and the microbe functional similarity matrix, we have constructed a unified matrix $H\in R^{\left(n_{y}+n_{w}\right)\times \left(n_{y}+n_{w}\right)}$ .


(8) $$H=\left[\begin{array}{cc} Y & A\\ A^{T} & W \end{array}\right]$$

where $A^{T}$ represents $A^{\prime }$ s transposition. As Equation 8 shows.

#### Predicting potential microbe–drug associations by BANs

Bilinear attention networks (BANs) are composed of a model proposed by Kim et al. (2018). The central component of BANs is the bilinear attention mechanism, which was initially designed to learn the distribution of attention by taking into account the bilinear interactions between the input channels.

In BANs, two pivotal technologies are used to enhance the interaction of features and manage intricate data relationships: bilinear transformation and attention mechanism. The bilinear transformation uses a weight matrix and an additive bias to process input features. It excels at revealing the nuanced relationships within complex datasets, providing a robust framework for analyzing interactions. The attention mechanism is a fundamental technique in neural networks, designed to improve the model’s focus on specific aspects of the input data. In the context of BANs, this focus is achieved through the application of bilinear transformations. These transformations provide a more adaptable way to adjust the weights associated with different features, thereby enhancing the model’s ability to prioritize relevant information within the data. Its formula can be expressed as:


(9) $$z=h^{T}\mathrm{Wh}+b$$

In the above Equation 9, h is the input vector of BANs, W is a trainable weight matrix, b is the bias term, and z is the output vector of BANs.

The forward propagation process of BANs is as Equation 10:


(10) $$x=W_{1}h+b_{1}x_{\mathrm{Relu}}=\mathrm{Relu}\left(z\right)=\left(x_{\mathrm{Relu}}^{T}Wx_{\mathrm{Relu}}\right)+bq=W_{2}z+b_{2}$$

where $W_{1}$ is the weight matrix of the first fully connected layer, $W_{2}$ is the weight matrix of the classification layer, $b_{2}$ is the weight matrix of the classification layer, q is the final output of the model, Relu is the activation function, defined as shown in Equation 11 and $x_{\mathrm{Relu}}$ is the feature vector processed by the Relu activation function.


(11) $$\mathrm{Relu}\left(h\right)=\{\begin{array}{c} h,h>0\\ 0,\mathrm{otherwise} \end{array}$$

Incorporating the BANs into predictive models enables a more nuanced capture of both the local features and the overarching structure of the data. This enhanced understanding, in turn, bolsters the model’s capacity for representation and elevates its predictive accuracy.

Obviously, after inputting H into the BANs, a low-dimensional matrix $Q=\left[\begin{array}{c} Q_{y}\\ Q_{w} \end{array}\right]\in R^{\left(n_{y}+n_{w}\right)\times l}$ can be derived, in which, the indices $Q_{y}$ and $Q_{w}$ represent the drug nodes and microbial nodes, respectively.

Thereafter, by integrating the drug matrix $Q_{y}$ , $Q_{w}$ with $W_{F}$ , and A separately inspired by Xuan et al., 2020, it is easy to see that we can construct a new drug feature matrix $R_{y}$ and a new microbe feature matrix $R_{w}$ as Equations 12, 13:


(12) $$R_{y}=\left[Q_{y},Y_{F},A\right]$$

(13) $$R_{w}=\left[Q_{w},W_{F},A^{T}\right]$$

Finally, based on $R_{y}$ and $R_{y}$ , we can obtain predicted scores for any given microbe $w_{j}$ and drug $y_{i}$ as follows:


(14) $$M_{ij}=\mathrm{Relu}\left(R_{y}\left(y_{i}\right).R_{w}{\left(w_{j}\right)}^{T}\right)$$

Hence, based on the above Equation 14, we can obtain a novel matrix *M*_1_ = [ $M_{ij}$ ].

#### Predicting potential microbe–drug associations by NNM

The kernel norm, alternatively referred to as the Schatten *p*-norm, is a matrix norm characterized by its reliance on the singular values of the matrix in question (Recht et al., 2010). This concept is pivotal in the field of optimization, particularly in the context of kernel norm minimization (Candès and Recht, 2012). The essence of this technique lies in reducing the kernel norm of a matrix, which is essentially the aggregate of its singular values. By doing so, it becomes feasible to approximate solutions for matrices that exhibit low-rank properties.

In BANNMDA, we define the kernel norm of the prediction matrix E as Equation 15:


(15) $${\left\|E\right\|}_{*}=\overset{\min\left(m,n\right)}{\underset{i=1}{\sum }}\sigma_{i}\left(E\right)$$

where ${\left\|E\right\|}_{*}$ denotes the nuclear norm of the matrix E , $\sigma_{i}\left(E\right)$ is the *i*-th largest singular value of the matrix E , and m and n are the number of rows and columns of the matrix E , respectively.

The objective of minimizing the nuclear norm is to identify a matrix E that achieves the lowest possible nuclear norm value, subject to fulfilling specific constraints. The optimization problem can be mathematically formulated as Equation 16:


(16) $$\min_{E}{\left\|E\right\|}_{*}\;\mathrm{subject to}\;E_{ij}=A_{ij},\left(i,j\right)\in \Omega$$

Consider Ω as a set that encompasses the known positions of the elements. To ensure that the prediction results fall within the range of 0 to 1 and to enhance the model’s robustness against noise in the data, we impose the following constraints on the model, as Equation 17 shows:


(17) $$\min_{E}\|E\|{}_{*}\;\mathrm{subject to}\;∥\sigma_{\Omega }\left(E\right)-\sigma_{\Omega }\left(H\right)∥<\varsigma$$

In this context, ς denotes the measurement noise, which accounts for the random variations or inaccuracies in the data. Meanwhile, $\sigma_{\Omega }$ signifies an orthogonal mapping that is applied to Ω . Subsequently, we replace the inequality-constrained models with regularized ones.


(18) $$\min_{E}{\left\|E\right\|}_{*}+\frac{\omega }{2}{\left\|\sigma_{\Omega }\left(E\right)-\sigma_{\Omega }\left(H\right)\right\|}_{F}^{2}$$

where ω is the regularization parameter. Inspired by Huttner et al. (2020), we use enhanced Lagrangian functions and the alternating direction method of multipliers (ADMMs) to address optimization problems that incorporate equality constraints. Equation 18 can be rewritten into the following form:


(19) $$\begin{array}{c} {Ι}_{\gamma }\left(E,Y,Z\right)=\min_{E}{\left\|E\right\|}_{*}+\frac{\omega }{2}{\left\|\sigma_{\Omega }\left(E\right)-\sigma_{\Omega }\left(H\right)\right\|}_{F}^{2}\\ +\frac{\gamma }{2}{\left\|E-Z\right\|}_{F}^{2}+Y,E-\mathrm{ZKAY} \end{array}$$

where Y is an introduced auxiliary variable, Z is the Lagrange multiplier matrix, and γ>0 is the penalty parameter. The ADMM algorithm can solve E , Y , and Z iteratively, and in each round of iteration, there are the following three steps:


(20) $$E^{k+1}=\mathrm{argmin}\left(\frac{\omega }{2}{\left\|\sigma_{\Omega }\left(E\right)-\sigma_{\Omega }\left(H\right)\right\|}_{F}^{2}+\frac{\gamma }{2}{\left\|E-Z\right\|}_{F}^{2}+Y,\;E-Z\right)$$

(21) $$Y^{k+1}=\mathrm{argmin}\left({\left\|E\right\|}_{*}+\frac{\gamma }{2}{\left\|Y-E^{k+1}+\frac{1}{\gamma }Y^{k}\right\|}_{F}^{2}\right)$$

(22) $$Z^{k+1}=Z_{k}+\gamma \left(E^{k+1}-Y^{k+1}\right)$$

Obviously, based on the above Equations 20–22, after *k* rounds of iteration, we can finally obtain a convergent matrix E , in which, the unknown values in A have been completed.

#### Calculating the final predicted scores of potential microbe–drug associations

In this section, we will use a weighted average approach to amalgamate the outcomes of the two prediction models. This method assigns different weights to each prediction, reflecting their relative importance or reliability. By doing so, we can create a composite forecast that leverages the strengths of both models while potentially mitigating the weaknesses of either.

The final microbe–drug associations prediction score matrix *M* is calculated as follows:


(23) $$M=\alpha M_{1}+\left(1-\alpha \right)E$$

where 0 ≤α≤1 is the weight value.

#### Model evaluation method

To enhance the model’s generalization capability and robustness, and to ensure the stability and reliability of performance evaluation, we implemented a five-fold cross-validation to assess the model’s predictive performance. Initially, we randomly selected 80% of the recognized and unrecognized associations from the dataset as the training dataset, while the remaining 20% was the independent testing dataset. Subsequently, we further randomly divided the training dataset, which was derived from the full dataset, into five equally sized subsets to facilitate the five-fold cross-validation. By utilizing the MDAD dataset, we performed five separate cross-validations, while ensuring that each trial was conducted independently. Upon the completion of the five-fold cross-validation, the model’s performance was assessed across various subsets of the training set. Ultimately, we used the pre-allocated independent test set to evaluate the model’s final performance.

## Experiments and results

In this section, we first conducted a sensitivity analysis of key parameters to optimize the model’s performance. Then, we selected six leading-edge methods for comparison with BANNMDA. To further validate the reliability of our model, we specifically chose two representative microbes and drugs for testing.

### Parameter sensitivity analysis

**Figure 2:**
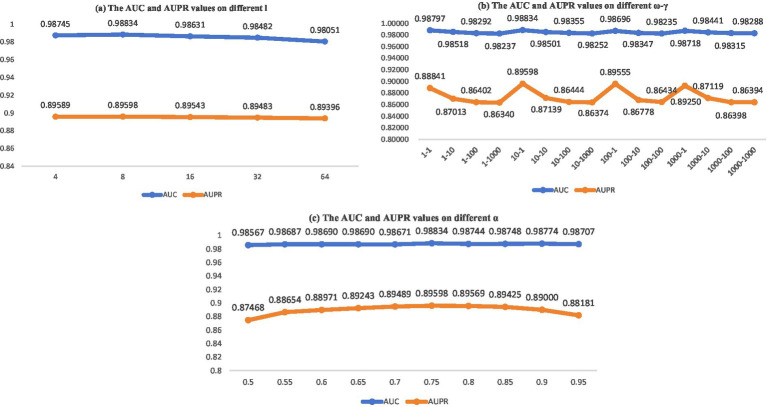
AUC and AUPR values on different parameter sensitivity analysis.

Considering the actual conditions of the model, we identified and analyzed four parameters that significantly impact the final predictive outcomes. In this context, within the BANs, dimension l emerges as a pivotal parameter. Within the NNM, parameters ω and γ specified in Equation 19 hold significant importance. In Equation 23, parameter α stands out as another crucial element. In this part, we aimed to identify optimal settings and maintain the separation of our training and testing datasets. In BANs, we resolved to modify the dimensionality parameter l , which was initially derived from the set $\left\{4,8,16,32,64\right\}$ . Subsequently, using a five-fold cross-validation (CV) approach, we assessed the area under the receiver operating characteristic curve (AUC) and the area under the precision-recall curve (AUPR) for the parameter configuration. The results are presented in Figure 2A.

In NNM, we opted to perform comprehensive tests by adjusting parameters ω and γ , derived from $\left\{1,10,100,1000\right\}$ , and carried out integrated experiments. The results are presented in Figure 2B.

Ultimately, the outcomes are presented in Figure 2C, which illustrates the influence of parameter α in Equation 23 after its modification from $\left\{0.50,0.55,0.60,0.65,0.70,0.75,0.80,0.85,0.90\right\}$ within the context of a five-fold CV on the MDAD dataset. The parameter analysis is depicted in Figure 2. As illustrated by the data in Figure 2, the optimal model performance is attained when the parameters are configured as follows: ω = 10, γ = 1, l = 8, and α = 0.75.

### Comparison with advanced methods

To enhance the validation of BANNMDA’s predictive capabilities, this section presents a comparative evaluation against six notable and competitive methods. During experiments, we adopted the original parameters of each competing method and executed all competitive methods using the same five-fold cross-validation approach on the MDAD dataset to ensure a fair and consistent comparison.

- HMDAKATZ (Zhu et al., 2019): The method harnesses the KATZ algorithm as its foundation to predict associations between microbes and drugs.
- SCSMDA (Tian et al., 2023): This approach uses a structure-enhanced contrastive learning technique coupled with a self-paced negative sampling strategy to forecast associations between microbes and drugs.
- GSAMDA (Tan et al., 2022): This model utilizes graph attention networks and sparse autoencoders to provide a new approach for predicting potential microbial drug interactions.
- GACNNMDA (Ma et al., 2023): Incorporating graph attention networks alongside CNN binary classifiers, this model pioneers a novel predictive framework for identifying potential microbial drug interactions
- GARFMDA (Kuang et al., 2024): This model deduces potential associations between microbes and drugs through an integration of graph attention networks and a dual-layer random forest architecture.
- MDASAE (Fan et al., 2023): This model uses a stacked autoencoder along with a multi-head attention mechanism to extract and understand the complex association system between microbes and drugs.

**Figure 3:**
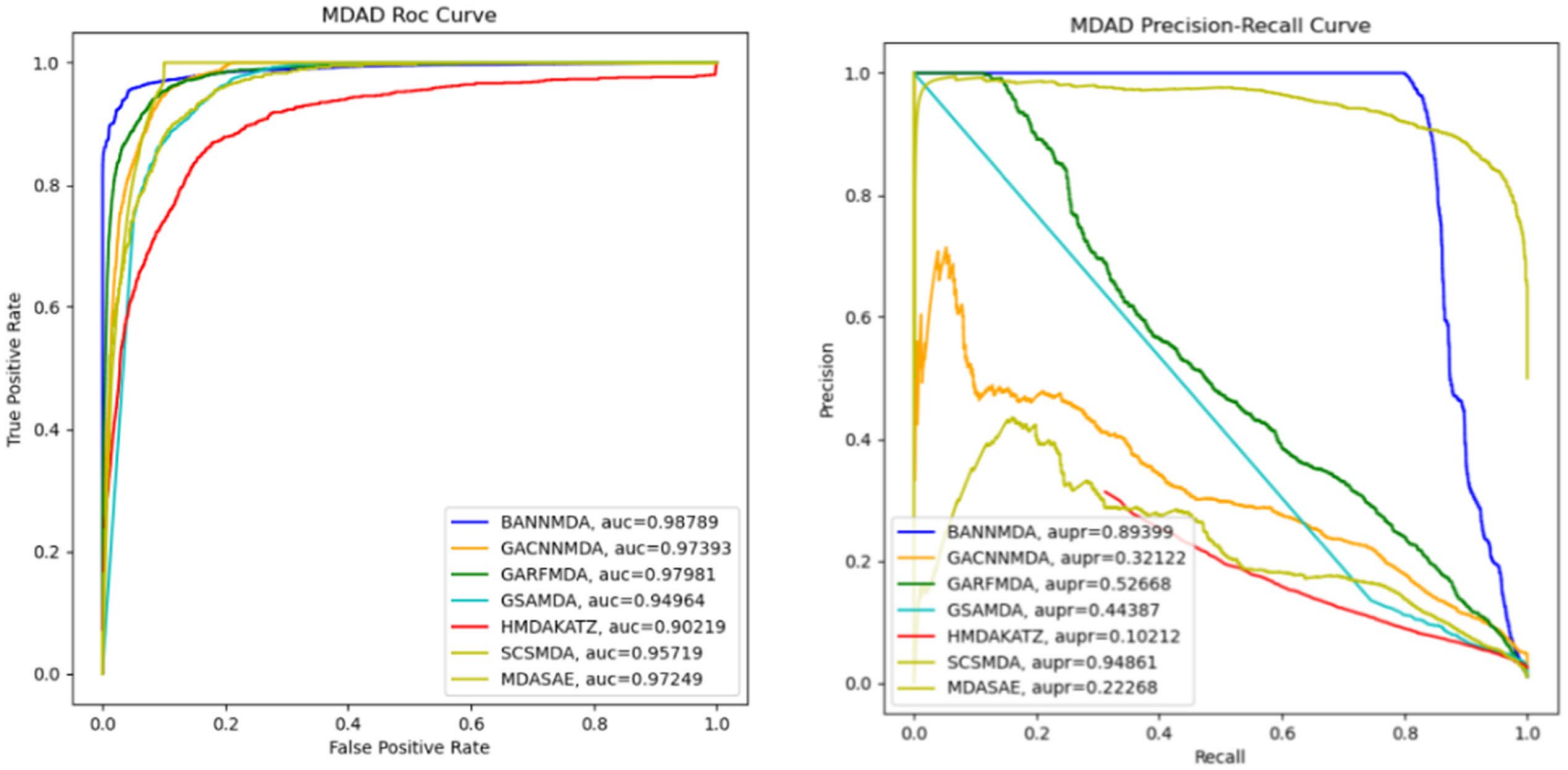
AUC and AUPR curves of six competitive methods based on the MDAD dataset.

We performed an assessment of these techniques with their default parameters and measured their performance via a five-fold CV process. The efficacy of the introduced BANNMDA model was evaluated using the AUC, AUPR, accuracy, and F1-score metrics, utilizing the MDAD dataset. The findings are detailed in Table 2 and Figure 3, showcasing the BANNMDA model’s exceptional predictive accuracy, surpassing the other evaluated approaches.

**Table 2:** Results of the compared methods.

Methods	AUC	AUPR	Accuracy	F1-score
HMDAKATZ	0.9012 ± 0.0013	0.1011 ± 0.0071	0.9774	0.3551
SCSMDA	0.9566 ± 0.0037	**0.9478 ± 0.0059**	0.9885	0.7016
GSAMDA	0.9462 ± 0.0017	0.4428 ± 0.0011	0.9896	0.6433
GACNNMDA	0.9783 ± 0.0015	0.3153 ± 0.0311	0.9944	0.7092
GARFMDA	0.9739 ± 0.0021	0.5189 ± 0.0213	0.9957	0.7103
MDASAE	0.9611 ± 0.0021	0.2282 ± 0.0013	0.9879	0.6957
BANNMDA	**0.9883 ± 0.0014**	0.8959 ± 0.0012	**0.9979**	**0.8893**

The bold values are the maximum values of each column.

As shown in Table 2, our model excelled in three of the four assessment criteria, with only a slight lag behind the SCSMDA model in the AUPR metric. The lower AUPR value compared to the SCSMDA method may be attributed to the SCSMDA method’s use of a self-paced negative sampling strategy, which adeptly selects negative samples that are richest in information content for training purposes. This approach is particularly effective in addressing imbalanced datasets and consequently elevates the AUPR values. Consequently, BANNMDA stands out as a highly effective predictive tool.

### Case study

To rigorously evaluate the predictive capabilities of the BANNMDA model, we selected two renowned drugs—amoxicillin and ceftazidime—as well as two prevalent microbes—*Bacillus cereus* and influenza A virus—for our case studies.

Amoxicillin (Huttner et al., 2020), classified within the penicillin family of antimicrobials, has been the subject of numerous studies that have demonstrated its association with the activity against *Bacillus subtilis* (Matei-Lațiu et al., 2023), *Clostridium perfringens* (Sárvári et al., 2022), and *Listeria monocytogenes* (Sixt et al., 2024). Based on the predictive scores, the microbes related to amoxicillin were ranked in descending order of their scores. After excluding the three associations already present in the MDAD dataset, the top 10 microbes were selected for further validation. As shown in Table 3, of the top 10 predicted microbes associated with amoxicillin, nine have been confirmed by existing research indexed in PubMed. For instance, Dewachter et al. (2022) confirms that amoxicillin has antibacterial effects against *Streptococcus pneumoniae*, while Gómez-Sánchez et al. (2023) establishes the association between amoxicillin and *Staphylococcus aureus*.

**Table 3:** Top 10 amoxicillin-associated candidate microbes on MDAD.

Microbe	Evidence
*Streptococcus pneumoniae*	PMID: 35748540
*Escherichia coli*	PMID: 33581330
*Staphylococcus aureus*	PMID: 36099212
*Halomonas pacifica*	Unconfirmed
*Haemophilus influenzae*	PMID: 32585694
*Pseudomonas aeruginosa*	PMID: 31026042
*Escherichia coli* O6:H1	PMID: 31777977
*Micrococcus luteus*	PMID: 8842345
*Staphylococcus epidermidis*	PMID: 27491399
*Streptococcus mutans*	PMID: 24423468

Cefotaxime is a potent aminothiazolyl cephalosporin antibiotic, renowned for its efficacy against a spectrum of Gram-negative bacteria (Gentry, 1985). Multiple research studies have highlighted the effectiveness of cefotaxime, showing its association with combating infections caused by *Pseudomonas aeruginosa* (Wang et al., 2023), *Escherichia coli* (Feng et al., 2021), *Streptococcus pneumoniae* (Ataee et al., 2014), and various other pathogens. As detailed in Table 4, following the exclusion of seven known associations recorded in the MDAD dataset, we identified nine microbes from the top 10 predicted cefotaxime-associated microbes that have been substantiated by PubMed-indexed literature. For instance, Awad et al. (2014) examined the relationship between cefotaxime and *Staphylococcus aureus*.

**Table 4:** Top 10 cefotaxime-associated candidate microbes on MDAD.

Microbe	Evidence
*Staphylococcus aureus*	PMID: 24723282
Enteric bacteria and other eubacteria	PMID: 3902652
*Acinetobacter baumannii*	PMID: 32043433
*Francisella novicida*	Unconfirmed
*Haemophilus influenzae*	PMID: 19803011
*Aggregatibacter actinomycetemcomitans*	PMID: 28668698
*Bacillus subtilis*	PMID: 31420587
*Staphylococcus epidermidis*	PMID: 1730894
*Streptomyces* sp.	PMID: 25737024
*Mycobacterium tuberculosis*	PMID: 28875168

*Bacillus cereus*, a Gram-positive bacterium characterized by its rod-shaped structure and beta-hemolytic activity, is frequently detected in soil and food products. This organism is notorious for its role in foodborne illnesses, particularly the “fried rice syndrome,” a form of food poisoning (Leong et al., 2023). Based on the pertinent literature, there is confirmation of associations between *Bacillus cereus* and various substances, including copper sulfate (Arokiyaraj et al., 2019) and silver nitrate (Babu et al., 2011). Upon the exclusion of three known associations recorded in the MDAD dataset, an analysis of the top 10 predicted drugs associated with *Bacillus cereus* identified 8 that have been confirmed by studies indexed in PubMed, as presented in Table 5. Park et al. (2022) elucidates the association between *Bacillus cereus* and rifampicin through an investigation into the prevalence and traits of toxin-producing *Bacillus cereus* strains isolated from low-moisture foods.

**Table 5:** Top 10 *Bacillus cereus*-associated candidate drugs on MDAD.

Drug	Evidence
Zinc sulfate	PMID: 4990588
Rifampicin	PMID: 36278133
Epigallocatechin gallate	PMID: 28941901
LL-37	PMID: 16801407
Vancomycin	PMID: 38785365
Toremifene	Unconfirmed
Curcumin	PMID: 26026869
Farnesol	PMID: 37717394
Dispersin B-KSL-W wound gel	Unconfirmed
Indole	PMID: 36869296

Influenza A virus is a member of the Orthomyxoviridae family, renowned for its significant pathogenic potential in humans (Nypaver et al., 2021). Existing scholarly studies have documented associations between the influenza A virus and a range of pharmaceuticals, including ribavirin (Ayari et al., 2021), zanamivir (Lee et al., 2022), oseltamivir (Ormond et al., 2017), and others, highlighting their potential roles in treatment strategies. Upon the exclusion of five known associations from the MDAD dataset, Table 6 reveals that nine out of the top 10 candidate drugs identified were correlated with the influenza A virus, underscoring a significant connection. For instance Li et al. (2022) highlights the significant role of curcumin in inhibiting the influenza A virus.

**Table 6:** Top 10 influenza A virus-associated candidate drugs on MDAD.

Drug	Evidence
Curcumin	PMID: 36365240
Epigallocatechin gallate	PMID: 33829450
Vancomycin	PMID: 16648946
Ciprofloxacin	PMID: 24400794
LL-37	PMID: 25082153
Betulin	PMID: 12837369
Toremifene	PMID: 30700611
Farnesol	PMID: 33811524
Azithromycin	PMID: 31300721
IDR-1018	Unconfirmed

In summary, these pairs of case studies provide additional evidence of the BANNMDA model’s capability to predict potential associations between microbes and drugs.

## Discussion

The linkage between drugs and microbes is of pivotal significance in the therapeutic realm of disease management, as emphasized by biomedical inquiries. Therefore, the advent of a sophisticated computational model for predictive models can significantly bolster the discovery of novel microbe–drug associations, optimizing treatment modalities for a spectrum of diseases.

In this study, we introduced a novel model BANNMDA by integrating the BANs and NNM to detect potential associations between microbes and drugs. The BANNMDA model was initiated by amalgamating the drug similarity network with the extant microbe–drug associations, alongside the similarity and association data between the nodes, to construct a novel heterogeneous network for microbes and drugs. Subsequently, the model leveraged both the BANs and the NNM to prognosticate the correlation scores between these microbes and drugs. To derive the predictive outcomes, these two forecasted scores were averaged with assigned weights. The empirical results demonstrated that BANNMDA surpassed contemporary methodologies and yielded satisfactory results in case study evaluations.

Although the BANNMDA model offered commendable predictive performance, there was still room for improvement. Notably, the BAN component of the model, while proficient in assimilating diverse information across heterogeneous networks, has demonstrated limitations in capturing the subtleties of local neighborhood information. This limitation is crucial as local neighborhood information is pivotal for understanding the intricate relationships within complex networks. The BAN model’s limitation in this area may be due to its inability to fully explore the nuanced interactions between nodes and their immediate surroundings, which is required for accurate predictions in network-based tasks. To address this, integrating BANs with graph convolutional networks (GCNs) could be a strategic approach. GCNs are particularly adept at leveraging local neighborhood information by aggregating features from neighboring nodes, which can significantly enhance the model’s representational capabilities. This fusion would allow for a more comprehensive understanding of the network’s structure and the relationships between nodes, leading to improved predictive performance.

Furthermore, to elevate the precision of the model’s forecasts, the incorporation of an expanded array of biological data was suggested. This enrichment would involve incorporating comprehensive data on drug side effects, elucidating the ties between bacterial strains and diseases, and exploring the linkages between pharmaceuticals and disease pathology. By doing so, the model gains a more intricate and detailed understanding of drugs and microorganisms, thereby improving the accuracy of its predictions.

## Conclusion

In conclusion, the BANNMDA model presents a significant advancement in the field of computational prediction of microbe–drug associations. It has demonstrated superior performance compared to existing methods, as evidenced by its successful application in case study evaluations. However, the model’s predictive capabilities can be further enhanced by integrating graph convolutional networks (GCNs) to better capture local neighborhood information and by expanding the scope of biological data considered. This would provide a more nuanced understanding of the complex interactions between drugs and microbes, ultimately leading to more accurate predictions and a deeper insight into disease management. The future incorporation of these enhancements is anticipated to propel the BANNMDA model to new heights in its predictive accuracy and applicability in therapeutic strategies.

## Data Availability

The original contributions presented in the study are included in the article/supplementary material, further inquiries can be directed to the corresponding authors.
